# On the implications of desexualizing vaccines against sexually transmitted diseases: health policy challenges in a multicultural society

**DOI:** 10.1186/s13584-017-0153-4

**Published:** 2017-07-01

**Authors:** Baruch Velan, Yaacov Yadgar

**Affiliations:** 1 The Gertner Institute for Epidemiology and Health Policy Research, Sheba Medical Center, Tel Hashomer, Ramat-Gan, Israel; 2 Department of Political Studies, Bar-Ilan University, Ramat-Gan, Israel

**Keywords:** Vaccination, Sexually-transmitted-diseases, Multicultural-society bioethics, Health-promotion, Hepatitis B Virus (HBV), Human Papilloma Virus (HPV)

## Abstract

Two vaccines against sexually transmitted infections are included in many national vaccination programs: Hepatitis B Virus (HBV) vaccine and Human Papilloma Virus (HPV) vaccine. The trajectories of the implementation of these two programs were marked by differences in the way the sexual context of risk was communicated to the public. These trajectories fluctuated between full accounts of the sexual nature of the infection and attempts to desexualize the vaccines. Vaccine desexualization can be achieved by withholding information of sexual context, blurring information, and distancing the age of vaccination from the age of sexual debut. Desexualization may be advantageous in promoting public health and personal health of people who believe that HPV vaccination leads to increased promiscuity, people who believe that protection against STD is not relevant to their children, and people who are not comfortable discussing the sexuality of their children. On the other hand, desexualizing may be disadvantageous for children to parents who tend to express passiveness towards vaccination, parents who attribute importance to sex education, and teenagers with homosexual orientations. The ethical analysis of vaccine desexualization reveals a complex interplay of considerations related to utility, causation of harm, duty of transparency, right to know, and right not to know. This analysis suggests that the moral merits of applying desexualization are questionable. Lastly, a sociopolitical consideration of the matter, suggests that decisions on vaccine desexualization can have implications on the interrelationships between various social groups and subgroups composing a certain population, and may highlight intercultural schisms. All this indicates that shaping the sexual framework of vaccination programs bears implications far beyond the practical considerations of vaccine promotion.

## Background

Promoting vaccine acceptance and advocating for healthy sexual behavior are two of the more challenging tasks of health authorities nowadays. Compliance with vaccination is compromised by growing hesitancy [1, 2] increased skepticism [3, 4] and exposure to anti vaccination movements [5, 6]. Promotion of healthy sexual behavior requires addressing the complexities, sensitivities and cultural disparities related to human sexual behavior [7, 8]. Vaccination programs against sexually transmitted infections by viruses such as Human Papilloma Virus (HPV) and Hepatitis B Virus (HBV) bring together the two demanding tasks of promoting vaccination and promoting sexual health, thereby doubling the confrontation. This challenge becomes even more complicated with the recent introduction of HPV vaccination targeted at adolescents at the awakening of their sexual development. In order to overcome the notable hurdles of promoting HPV vaccination, policymakers could be tempted to downplay the sexual implications of this health intervention and to desexualize the vaccine.

In this article we shall examine the processes of desexualization. We shall denote desexualization as the narration and (re)presentation of the diseases, viruses and vaccinations as unrelated to sex or sexual activity. We shall demonstrate that the public discourses, narratives and representations constructed around a certain object (disease, virus, and vaccine) can either highlight its relation to sex, explicating the virus spreading via sexual activity, or instead downplay, to the point of only implying this connection. Alternatively, as we shall demonstrate, desexualization can take place by applying the vaccine in pre-adolescence age, so as to detach if from the “sexualized” ages of adolescence and adulthood. In this way, desexualization leads to a shift of focus away from the mode of infection – that is, the manner in which the virus is spread, and instead a highlighting of the disease caused by the virus.

We shall demonstrate that desexualization may be encouraged by several considerations. As the case histories we outline below show, these may include an attempt to avoid stigmatization of certain minority groups in society, or to overcome opposition to vaccination that is nourished by parents’ uneasiness or even unwillingness to consider the (present or future) sexual activity of their children. In both cases, desexualization entails a downplaying, or even hiding, of certain information – such that would be seen as “uncomfortable” to handle or as holding the potential to stigmatize certain groups – for the sake of achieving a successful epidemiological result.

We would argue that while the tactic of desexualization may seem tempting for policy makers as a means for neutralizing the opposition to vaccination in certain cultural groups offended by sexual context, the disadvantages that desexualization entails may outweigh its benefit. This, as we shall argue, touches upon both ethical matters and social ones.

### Desexualization trajectories

Two vaccines against sexually transmitted infections are currently included in most of the vaccination programs in developed countries: Vaccination against Hepatitis B Virus (HBV), first introduced in 1982, and vaccination against HPV, introduced in 2006.

The trajectories of the implementation of these two programs are marked by shifts in the way the sexual context of the risk were communicated to the public [for a comprehensive review see [9]). In both cases, communication fluctuated between full accounts of the sexual nature of the infection and various attempts to desexualize the vaccine. The dynamics of these fluctuations differed from one program to the other. The two programs were affected by the different cultural atmospheres at the time of vaccine introduction, as well as by the major differences between the natural histories of HBV and HPV infections. This turned out to ease the desexualization of HBV infection, yet hamper attempts to desexualize HPV infections. Our discussion will deal mostly with case histories from the US and Israel, but we will also consider certain aspect of other case histories, which may illuminate our discussion.

### Vaccination against HBV

Hepatitis B is a viral infection that attacks the liver and can cause an acute and a chronic liver disease, and even liver cancer. Hepatitis B is transmitted by exposure to infected blood and various body fluids, such as saliva, menstrual, vaginal, and seminal fluids [10]. Hepatitis B is spread either through vertical perinatal transmission (from mother to child at birth), or through horizontal transmission (exposure to contaminated blood and body fluids). Horizontal transmission can occur by casual contact among children during the first 5 years of life, as well as among healthcare workers handling infected blood, among people who inject drugs and among individuals who practice unsafe sex [10]. The relative incidence of these infections differ from country to country, vertical transmission being more common in some regions of the world and horizontal transmission in others [11]. In the USA, for example, the most frequently reported risk factors for acute hepatitis B in 1990 were heterosexual activity (41%), injection drug use (15%), homosexual activity (9%), household contact with a person with hepatitis B (2%), and healthcare employment (1%) [12].

Tracing the history of HBV vaccination reveals a notable shift from a rather sexualized program in the early 80s to a desexualized program nowadays. The first generation HBV vaccine (introduced in 1982) was produced by isolating viral antigens from the blood of infected people [13]. This vaccine was targeted to populations at risk, identified as health professionals, selected immigrants, injection drug users, sex workers and gay men. The use of HBV vaccines as a universal vaccine in the general population was trumped at this stage by the belief that unlike other childhood vaccines, HBV is slow in spreading and is not of an epidemic potential. In addition, the fact that this vaccine was a blood product made it less amenable to production in large scales required for routine vaccination programs. Moreover, injectable biologicals originating from human blood invoke safety considerations, especially if this blood is associated with risky behaviors of the donors. These perceptions of risks were boosted by the emergence of the AIDS epidemic in the early 80s and the tagging of both HIV and HBV as “gay infections” [9].

This led to a conceptual linkage between sex, infection, risk, and stigmatized groups, a linkage which had the potential of endangering the future HBV vaccination, as well as the future of therapy based on immunoglobulin, yet another blood-derived product. It should be noted however, that the highly-sexualized HIV/HBV linkage had also a positive outcome, namely the contribution of increased awareness of safer sex practices and condom use to hepatitis containment [14].

The development in 1986 of a new generation of safer recombinant HBV vaccines, produced commercially in yeasts, set the stage for a desexualization process: losing the identity of HBV as a sexually STD agent and adopting an identity of a cancer-causing agent [9]. This entailed the shift of focus from the mode of HBV transmission to the clinical outcomes of the infection. Emphasis was put on the dangers of acquiring chronic liver disease upon infection at early age, and the associated risks of developing cirrhosis and liver cancer. The new HBV vaccine was now tagged as a universal vaccine for infants [13], leading to a gradual process of disentanglement of HBV from stigmatized identities and implementation of general use.

At present, HBV vaccine is targeted to the general population in many developed countries including the USA and Israel. Nevertheless, in other countries, including the UK and Japan the prevalence of the diseases does not justify universal vaccination, and the HBV program is targeted to a list of specific groups at risk. In the UK the list specifies “people who change their sexual partners frequently” and men who have sex with men (MSM) [15].

In Israel the introduction of universal HBV childhood vaccination in 1992was driven by the influx of new immigrants from two highly endemic regions, the former USSR and Ethiopia, and the realization that newborns of these immigrants are at high risk of chronic hepatitis [16]. At these early years, HBV vaccination in Israel carried the potential of dual stigmatizing, not only by tracing HBV to the female birth canal, but specifically to the birth canal of ostracized population groups. This led the Israeli policymakers of the time to focus on the disease rather than the mode of infection. The Israeli universal HBV vaccination policy turned out to be successful [17]: routine HBV vaccination at early childhood is not a subject of controversy in Israel, and coverage rates are high.

Thus, while epidemiologically HBV vaccine desexualization has proven to be efficient, it entails, in practice, a “marketing” of the vaccination that is based on downplaying, information that the consenting parents (agreeing to have their newborn child vaccinated against a virus that may be only rarely found in the social group to which the parents belong) would probably find relevant, were it suggested to them. To put it bluntly, the desexualization of the HBV vaccination in Israel allowed vaccination of a majority group for the prevention of stigmatizing certain minority groups.

### Vaccination against HPV

HPV infections are spread by a set of ~100 virus subtypes that colonize the human skin and mucosal surfaces. HPV vaccines are targeted against strains that infect the female and male genitalia as well as the anal and the oral cavities and can cause genital warts and cancer [18]. These strains are transmitted almost exclusively through sexual activity. Therefore, HPV vaccination programs are aimed at preventing cancer (mainly cervical cancer) by preventing the spread of a highly contagious sexual infection, and are, therefore, targeted at adolescents prior to their sexual debut [18].

The sexual context of HPV was harder to obscure when compared to HBV. This is related to the stronger linkage of HPV infection with sexual transmission, and to the fact that HPV vaccine introduction took place in the beginning of the 21^st^ century, when the public tends to be less likely to accept vaccine recommendations without questioning them [3, 19]. Based on the experience gained from HBV history, the developers of the HPV vaccine attempted to introduce their product to the US market without drawing attention to matters of sexual behavior [20].

The HPV vaccine was “packaged” and marketed as a vaccine against cancer rather than a vaccine against sexually transmitted infections (STI). The HPV vaccine was portrayed as the best approach to early cervical cancer prevention, through appropriate advertising campaigns, and by lobbying US legislators to introduce mandatory HPV vaccination [9, 21]. All these attempts to desexualize HPV failed to prevent a stimulated public debate related to HPV transmissibility and adolescent sexuality. It is interesting to note that the HPV vaccination rates of adolescent girls in the USA are relatively low (below 40%), compared to those in Australia and Northern European countries (~70 and ~80% respectively) [22]. This could be related to the fact that in Australia and the UK vaccines are given in schools, whereas in the US vaccination is in the domain of the private practitioner. Nevertheless, it could be also attributed to the US culture, which tends to be more puritan, as reflected by the abortion wars and the attitudes of certain groups to sex educations, contraception and abstinence.

In Israel a universal program for HPV vaccination of girls at middle school was introduced in 2013. Overall HPV coverage for girls is ~60%. Notably, coverage appears to be rather high among Israeli-Jews, who self-identify as “secular” (70–80%), and low in religious and Orthodox Jewish communities (0–35%) [Vaccination records of the Israeli MOH]. Interestingly, the compliance rates of the Arab population, which tends to be more conservative, against HPV are high and are comparable to those of secular Jews. This correlates with the tendency of this population group (Arab citizens of Israel) to adhere with the recommendations of the Israeli health authorities in general [3].

A recent twist in the sexualization-desexualization debate occurred when HPV vaccination was approved for adolescent boys and later for young males [23]. Profiling boy vaccination merely as a means for protecting girls against cancer appears to be a poor incentive for parents to vaccinate their sons [4]. This led to discussions on various aspects of HPV-related sexual infection in males, including discussing the rather stigmatizing appearances of penile warts [24] and the risks of anal cancer in men [25]. This in turn led to the reemergence of gay men as a specific risk group for sexually transmitted infection [26], and highlighted the health implications of anal sex among homosexuals as well as heterosexuals. Altogether, HPV vaccination programs of males contributed to the strengthening of HPV sexualization.

### Techniques for desexualization

Desexualization of vaccines against STD can be achieved by various techniques including: withholding information of sexual context, blurring it and emphasizing other means of transmission on the one hand and on the other hand, distancing the age of vaccination from the age of sexual debut. These techniques, when used separately or in combination, could be perceived as means for facilitating vaccine acceptance in certain population groups, by “neutralizing” opposition that may be driven by resistance to explicitly acknowledging aspect of human sexuality.

### Withholding information

Hiding all information related to the sexual nature of HPV infection is practically impossible. Nevertheless, an examination of formal communications issued by various health authorities on HPV vaccines reveals various levels of transparency. For example, full disclosure is provided by the Australian Department of Health [27]. The first statement in their guidelines leaves no doubt about the context of HPV infection:**“** *HPV is a common virus that affects both males and females, which is passed from person to person through sexual contact”*. This is followed by the statement “*HPV can cause cancer of the cervix, vulva, vagina, penis, anus, some head and neck cancers, and genital warts*”.

In contrast, the information provided by the South African health authorities [28] is aimed at withholding sexual information, mainly be leaving the matter implicit. This is exemplified the statements: “*More than 100 strains of HPV exist, and 30 of them are associated with below-the-belt cancer*”. This manifestation of HPV desexualization cannot be disconnected from the ongoing struggle of South America with a devastating AIDS epidemic, and with a deplorable attempts to desexualize HIV by South African politicians in the past [29].

An interesting modulation on desexualization is provided by the Columbian authorities. Here, the argument for vaccinating against HPV is defined by the statement *“The disease is very common among people younger than 25 years of age, since these are not aware of the dangers of HPV* [30]”. The fact that this age group is at risk because it engages in sexual activity is not mentioned.

Between these extreme approaches, one can find cases where only partial information is provided. The French communications [31] indicate that “*150 types of papilloma virus exist, 40 of which infect the genital organs of males and females*”, with no mention of oral and anal infections. Information on anal cancer in men appears to be very problematic and even the most transparent vaccination campaigns can be found guilty of obscuring this information. For example, the New Zealand MOH’s internet site dedicated to promoting HPV vaccination [32] is very direct about sexual transmission of HPV and the implication of sexual activity on HPV related morbidity in girls, yet remains obscure when it comes to morbidity in boys, stating that “*HPV infections in males also cause genital warts and a range of other HPV diseases*” not referring to oral and anal cancers acquired through sexual practice.

### Blurring information

The discourse on vaccines against STDs and the information it entails can be desexualized by coating the sexual content with nonsexual content. Manifestations of mild “coating” can be identified in some communication related to HPV vaccination. In the American and Israeli publications [33, 34] emphasis is given on the fact that HPV is a skin infection transmitted by skin to skin contact, thus sexual transmission is portrayed as a private case of skin contact. This information is correct, but can be confusing to the lay reader, and reduce alertness to the need to vaccinate prior to sexual debut. Another form of blurring sexual content is by stressing that “*One does not have to engage in sexual intercourse to get infected but intercourse increases the chance of infection*” [35]. This statement again is factually correct, and was probably introduced to warn against the potential infection risks from sexual foreplay, nevertheless this could be discursively misleading by diminishing the importance of sexual transmission of HPV.

### Distancing the time of vaccination from the time of sexual debut

The introduction of programs for newborn vaccination against HBV in the late 80s [10] turned out to facilitate the portrayal of the HBV vaccine as a general vaccine rather than a vaccine with sexual connotations. This cannot be the case for HPV vaccination: The lack of any medical indication for vaccinating very young children against HPV, together with the understanding that vaccination should begin prior to sexual activity, led policymakers to target HPV vaccination to boys and girls between the ages of 9–13 years [36]. In most countries HPV vaccination is conducted on youngsters at the higher age range of 12–13. Nevertheless, in 15 out of the 80 health systems practicing HPV vaccination, the program is targeted at the early age of 9 years [22].

Defining the age of HPV vaccination is a cultural consideration rather than a medical one. Vaccinating a 9 years old child would imply the vaccination of a naïve person, unable to understand the implication of the intervention exercised on her. In contrast, vaccination of a 13 year old, on the verge of puberty could constitute part of a coming of age process, where youngsters are prepared for adulthood and sexual responsibility. As a consequence vaccination at the age of 9 years should be perceived as an act of HPV desexualization, while vaccination at the age of 13 implies affirmation of the sexual context of the program.

Recently, a committee appointed by the Israeli MOH recommended changing the age of HPV vaccination from 13 years to 9 years of age [37], based on the assumption that this will facilitate the acceptance of the vaccine by the Jewish Orthodox community in Israel, given that in this population premarital sex and sometimes even premarital discussions related to sex are deemed unacceptable. This is a good example that lowering the age of vaccination against STDs is indeed perceived as a technique for desexualization.

In this context, it is interesting to note that HPV vaccination at the age of 9 years is practiced in the two African countries, South Africa and Angola, where national programs exist, as well as in most Latin American countries including Chile, Brazil, Mexico South Africa, Peru, and the Dominican Republic. This is opposed to the European countries (except Austria), the US, Australia and New Zealand were HPV vaccination is practiced at later ages. This may suggest a divide between states with traditional societies versus states with less traditional societies [22],

Interestingly, children in the Canadian province of Quebec are vaccinated at the age of 9, while in the province of Ontario the age of vaccination is 12–13 years [22]. This may be related to the fact that a large portion of Quebec’s population is Catholic. However, one cannot overlook the fact the Quebec’s legislation is often extremely secular and Quebec’s health policies are considered to be some of the most progressive in the Canada. All of this alludes to the complex role of cultural-political and religious factors in HPV vaccination policies.

These examples attest to the intricacy of vaccine desexualization. Mechanisms can differ in their character, in their directness and in the degree of desexualization. Needless to say, the varying cultural, historical, lingual and political contexts of the different cases discussed above necessitate a highly sensitive hermeneutical approach, which is largely absent from our interpretation above. Moreover, this article addresses desexualization as a uniform concept and does not attempt to examine separately the different manifestations of this concept – an undertaking that would surely demand more space than is allowed here.

The examples of desexualization presented above, relate to HPV vaccination programs, but are all applicable to desexualization of the HBV vaccine. HBV desexualization is deeply rooted in the history of HPV vaccination [see above in the chapter on “Desexualization Trajectories”], and is often associated with the multiple transmission modes of the virus. This allows for HBV desexualized by coating the sexual content with nonsexual content, as exemplified by the information provided by the Israeli MOH on HBV vaccine: “*The virus is transmitted in various ways: Contact with contaminated blood product, sexual interaction with a carrier, from an infected mother to the newborn at birth and close contact with an infected family member.”* This portrayal of HBV could be justified by the fact that sexual transmission is indeed only one of the ways of spreading the virus, but at the same time carries the danger of downplaying the significant role of sexual transmission in spreading of HBV.

### Putative advantages and disadvantages of desexualization

#### Advantages of desexualization

The advocates of desexualization would argue that the overt discussion on the sexual context of vaccination programs, such as that against HPV will deter certain populations from vaccinating their children, whereas desexualization will enhance compliance. Desexualization may be advantageous, from the point of view of promoting public and personal health, in the case of people who believe that HPV vaccination leads to increased promiscuity, people who believe that protection against STD is not relevant to their children, and people not comfortable discussing the sexuality of their children.

### Implications for parents who believe that vaccination will lead to increased promiscuity

Worries about promiscuity following HPV vaccination appear as a recurrent theme in qualitative studies. Parents from different backgrounds often express concerns that HPV vaccination will encourage sexual activity and serve as a carte blanche for behavior that might put youngsters at risk of unwanted pregnancy or contracting other STDs [38, 39]. Therefore, protagonist of HPV desexualization would argue that lowering the volume of the sexual discourse, will reduce parental worries and increase compliance.

Nevertheless, quantitative studies indicate that parents are less concerned about HPV vaccination as a mandate for engaging in sexual activity. Across 6 studies, only few parents (1–18%) expressed concern about the effect of vaccination on their child’s sexual behavior [40]. Moreover, empirical evidence does not indicate that HPV vaccination leads to dangerous sexual behavior. A study conducted on a very large cohort of Canadian girls (260,493 girls), found no evidence that HPV vaccination increased the risk of compromising outcomes such as teen pregnancy or STD infections [41].

### Implications for parents who believe that protection against STD is not relevant for their children

Some parents deny the fact that their adolescent children can be exposed to STDs. This could be related to psychological barriers of parents to accept the emerging sexuality of their children [42]. In addition, denial of adolescent sex may be embedded in the social norms of certain religious communities, where premarital sex is not perceivable [43, 44]. In such societies, HPV vaccination could be perceived as unnecessary, but more importantly, as an indication of moral flaws. Desexualization would thus allow for the vaccination of the youngsters without instigating their parents’ opposition on these grounds.

### Implications for parents who are not comfortable dealing with the sexuality of their children

Certain parents find it difficult to confront the idea that their young children are in the process of becoming sexual beings, and even more so that they might adopt sexual behaviors that do not conform to their own beliefs and standards. Some parents will find difficulties in initiating the direct ‘bees and birds’ discussion with their young children and try to circumvent it [45]. It would appear that liberal parents are more likely to discuss sex with their children than conservative or religious parents. Nevertheless, even the most outspoken parents could be uncomfortable discussing the dangers of unsafe oral or anal sex as well as the specific risks for men who have sex with men, all of which are related to HPV infections. The linkage between the need to talk to your child about sexual behavior and the need to explain HPV vaccination may result in postponing both the conversations as well as the decision to vaccinate, and ultimately in avoiding HPV vaccination altogether.

### Disadvantages from desexualization

The foreseen advantages of vaccine desexualization may be counterbalanced by several disadvantages. While, as suggested above, certain groups may gain from desexualization, others may lose from it. These include children to parents who tend to display passiveness towards vaccination, parents who attribute high importance to their role in sex education, as well as male teenager homosexual orientations. One would argue that an overt discussion on the sexual context of HPV would increase alertness for the need to get vaccinated, and serve as a boost for compliance in population groups, which are most prone to be infected.

### Implications for children to parents who tend to display passiveness towards vaccination

Previous studies have shown that passiveness has a significant role in noncompliance to vaccination [46]. Some individuals have indicated apathy, laziness and lack of interest, or just negligence as reasons for failure to get vaccinated. The bias of non-action can lead parents to stay at home and not vaccinate their children. A vibrant discussion on vaccination and sexuality appears to be a good remedy for apathy, whereas desexualization can increase unawareness and lack of action.

### Implication for parents who attribute high importance to their role in sex education

Many liberal parents assign importance to their role in the sexual education of their children, and may take advantage of HPV vaccination to discuss with their children the reason for being vaccinated in the general context of sexuality and responsible sexual behavior [47]. School vaccination in the 8th grade sets a defined time point for engaging in such a discussion and provides a good trigger for initiating the discussion [48]. Desexualization of HPV aborts this opportunity and has the potential of affecting the health of a child in all aspects related to sexual risk, in addition to those related to HPV.

### Implications for male teenagers with homosexual orientations

One of the less acknowledged aspects of HPV infections relates to the risk of anal cancer among MSM. Blocking the flow of this information may result in direct danger to the health of youngsters with homosexual tendencies. Most teenagers, as well as most parents do not acknowledge homosexual orientation [49]. Nevertheless, sexual orientation and tendencies start to develop at early age and in adolescents [50], and parents [51] are often aware of these developments. Therefore, it is important that information on the dangers associated with male homosexuality remains overt and that parents include such considerations in their vaccination decisions.

Examination of the putative advantages and disadvantages from desexualization reveals that certain groups in the population will gain from desexualization whereas others will lose from it. In other words, there is no basis for arguing that desexualization is “harmless”: while it may neutralize opposition to vaccination in certain segments of society, it may well also result in a decrease in participation in vaccination programs by individuals/families who would otherwise be alerted to the importance of vaccination against STDs. If we were to adopt a one-dimensional view of society as split across a secular-religious and modern-traditional axis (one dimensional and simplistic as this may indeed be), we may say that in most cases the religious and/or traditional groups will be on the gaining side resulting from demutualization, whereas the liberal, non-conservative or secular groups will be on the losing side.

It should be noted that gains and losses from desexualization of HBV can differ from those of HPV. One has to bear in mind that desexualization is an integral part of HBV portrayal in most Western countries, and the question to be asked is actually related to the effect of changing this *status quo*. It could be argued that a shift from a desexualized portrayal to a sexualized one will not affect people who are offended by sexual content. These will be comfortable with the argument that the virus is transmitted by non sexual contacts as well. On the other hand, awareness of the sexual content will encourage liberal parents to vaccinate their children against HBV to protect them from some the challenges of adult life.

### Ethical implications

Addressing sexuality in the context of HPV vaccination invokes ethical considerations of teleological and deontological nature [52]. The formers relate to desexualization in terms of consequence and utility, and the latters in terms of compliance with moral duties.

### Teleological consideration

The major argument for desexualizing vaccination against STDs is based on the assumption that it will enhance the utility of vaccination programs by boosting compliance among conservative or religious parents. The main objection to this assumption relates to the lack of quantitative empirical evidence. Moreover, desexualization can simultaneously lead also to decreased compliance among other groups in the population (see above), and balancing quantitatively these two effects is practically impossible.

In addition, withholding any information, including sex-related information may result in hurting the already fragile trust of the public towards health authorities in matters related to vaccination [53]. This in consequence can decrease the compliance of certain parents with other vaccination programs, and indirectly affect utility. All this suggests that the utility gained from desexualizing HPV remains speculative and questionable.

Harm related to sexualization-desexualization is two faceted. On one hand, refraining from desexualization may lead to morbidities among offspring of conservative parents who would not vaccinate their children because of their discomfort with the sexual connotations of HPV infections. On the other hand, practicing desexualization may lead to morbidities among offspring of liberal parents who fail to vaccinate their children because they are not fully aware of the need to vaccinate against HPV prior to sexual debut.

Balancing the potential harms inflicted on these two different populations is challenging. In lack of quantitative indication to the actual extent of the harm for the two parties, one has to resort to speculations. This will suggest that more harm can be caused by adhering to desexualization than by refraining from it. Young people from religious or conservative communities are more likely to follow a traditional lifestyle that opposes premarital sex and endorses monogamy, and are therefore less likely to be affected by HPV when not vaccinated. In contrast, youngsters in more permissive communities may be at greater danger to contract HPV if not vaccinated.

### Deontological considerations

Desexualization entails, almost by definition, an exercise of concealment, lack of transparency and even outright misinformation which may be seen as “conspiratorial” by the state [54]. Any attempt to withhold or manipulate information related to the sexual nature of HPV transmission or that on any other STDs is a clear breaching of transparency. In liberal-democratic nation-states, where the common perception sees the state as serving the benefits of its individuals, this amounts to a breach of trust. Furthermore, adopting a policy of desexualization could be considered as premeditated non-transparency. Premeditated non-transparency appears to be more contemptible than the act of not revealing information in the course of an ongoing process or *post factum*. While the latter is sometimes explained by negligence the former could be perceived as deception.

The state’s duty to be transparent is closely linked to the individual’s right to autonomy. Autonomy is defined as the right of patients to make decisions about their medical care without external influence [55]. Autonomic decisions depend very much on the information available to the lay individual. Obscuring information related to sexual aspects of a health measure is therefore an infringement on autonomy, and cannot be justified by paternalistic arguments: The prevalent sociopolitical contract in liberal democracies dictates that the state should not attempt to make people “more healthy” by hiding information.

In addition, HPV vaccination, targeted at youngsters aged 9–13 years becomes more complex as it invokes the issue of adolescent autonomy. While legally, children at this age are not considered to be autonomous, the involvement of an adolescent child in the decision to get vaccinated against a sexually transmitted disease should not be ignored [56]. Such an involvement is more meaningful as the child gets older. Therefore, desexualized vaccination programs targeted at 9 years old children undermine autonomy, whereas vaccination programs targeted at 13 years old promote it.

The right to know cannot be separated from the right not to know. Advocates of vaccine desexualization would argue that individuals may have a legitimate interest not to be informed about sexual contexts that may offend them, and that this interest may, in effect, constitute an enhancement of autonomy [57]. The emerging biomedical discourse tends to recognize the right of patients not to know in specific medical issues, including those related to genetic disorders and terminal illness. It is agreed, however, that nondisclosure, if exercised, should not pose any risk to the patient, that the right not to know cannot be presumed but must be “activated” by the individual’s explicit choice, and that this is not an absolute right, and should be restricted to specific cases [57].

Translating the right not to know from the personal level to the group level, as in the case of vaccine related information entails numerous problems. One cannot be sure that the request not to know is “activated” by all members of the group, and that it reflects a genuine request by the group. This becomes more acute in the case of sex-related knowledge, since here, the request not to know is often communicated by proxies, such a community leaders or figures from the religious establishment. In addition, the interrelationship between harm and knowledge is more complex in the group scenario: While one can argue that the knowledge about the sexual context of vaccines can deter some individuals in the group from vaccinating their children and generate harm, harm can also be inflicted to those within the group who seek information about the vaccine and don’t get it.

Finally, one could argue that the right not to know cannot be valid in any context related to transmittable diseases, since lack of knowledge can lead to a cascade of harm to others. One can hardly envision a situation where, once a HIV vaccine is available, explicit HIV-related sexual information would be withheld from persons or groups intimidated by this information.

### Balancing ethical considerations

Balancing between utility and duties in the context of vaccines against STD and more specifically HPV is complicated. Curbing fundamental duties for the sake of utility may be considered as justified when the risks of a given infectious disease are very high, but not justified in cases where risks are low. In Israel, for example, the epidemiological data on HPV do not indicate high urgency. The incidence of cervical cancer among Israeli women is relatively low (5.3 per 100.000 Israeli Jewish women and 2.3 per 100.00 non-Jewish women [58]) when compared to higher rates (15–40) in some regions in the world [59]. Furthermore, the incidence rate of abnormal PAP smears taken as a routine screening test among Orthodox Jewish women is very low (1.2%) and the vast majority of abnormal smears were later found to be false positive [60]. These findings cast doubt as to the need for comprehensive vaccination against the papilloma virus among low-risk populations. Moreover, this suggests that compromising autonomy and transparency through desexualization of HPV vaccination hardly justifies the potential gain of increasing compliance among Orthodox groups in Israel.

The ethical consideration related to HBV vaccination are somewhat different from those related to HPV vaccination. As in the case of HPV, the utilitarian effects of HBV sexualization remain questionable, since vaccine acceptance will probably not be effected by underlining the sexual context of HBV infection. On the other hand the deontological analysis reveals additional ethical aspects, related to the interplay between autonomy, state-paternalism and complexity. The complexity in addressing HBV vaccination stems from the wide array of modes for HBV infections, the varied considerations related to the age of vaccination, as well as the correlation between specific socio-demographic groups. All these complexities, which are guiding health professionals in their decision making, should not remain privileged information. The authorities should make the outmost efforts to communicate this complexity to a lay person that is asked to vaccinate her newly born child.

An additional approach for addressing the bioethical implications of vaccine desexualization is by examining it through the lens of Prinicipalism, focusing on autonomy beneficence nonmaleficence and justice [55]. As mentioned above, desexualsation constitutes a clear infringement on the principle of autonomy, which is often considered as first among equals when grading the four principles. Dissection of the attribution of desexualization to beneficence or nonmaleficence does not bring new insights; since the effects remain population-specific (see above the chapter on **“**Putative advantages and disadvantages of desexualization**”**). In contrast, analyzing vaccine desexualization in the context of justice brings a new perspective to the matter. One can claim that desexualization provides a “veil of ignorance” which serves justice. If no one is aware of the sexual connotations of HPV vaccination no one will judge the vaccine based on the sexual practices of a target population, but rather on its impact on cancer prevention. This in turn will lead to equity in the way different groups of the population address HPV vaccination programs and respond to it. The argument of the “veil of ignorance” proposed by John Rawls may be valid when addressing social injustice. Nevertheless, its implication in health issues is problematic, as it stands in contrast to the current emphasize on patient enhancement.

### Sociopolitical implications

While political culture in many Western countries upholds a rather uniform self-image of “the West” and “the Western”, sociopolitical reality clearly shows this to be a false image, which is confronted with a de-facto heterogeneity of identities. New social realities, resulting from, both immigration [61] and intensification and legitimization of internal diversities, challenge the unifying nation-statist impulse, which tends to view the “population” at large as one homogenous whole, with the “harsh” truth of sociocultural diversity.

Thus, diverse ethnicities, differing class status, and varying communal historical trajectories are manifested in disparate attitudes and practices, that far surpass a one-dimensional religious-secular, or modern-traditional dichotomy, which is prevalent in the Western discourse [62, 63]. Specifically, for the purpose of the current discussion, it is important to note that the dominant self-image of the West, instigated by the European Enlightenment and driven by the political, economic, and cultural force of the modern nation-state faces a reality of *multiple* modernities, which challenge many of what have come to be taken-for-granted truisms of “the West” [64].

A prevalent, yet clearly misleading conceptual scheme would have this as yet another expression of a supposed division of humanity along a “modern-traditional” axis. However, as we alluded to above, such a view fails to appreciate alternative understandings and constructions of modernity [65–67]. In any event, what is of urgent relevance to the matter discussed in this paper is the fact that a sociopolitical reality of diversity also impacts directly upon public health policies, including vaccination policies, demanding that the policy makers acknowledge the heterogeneity of the allegedly coherent notion of “population” to which they apply their policies. Thus, the design of any given national health policy requires a thorough examination of the cultural multiplicity of the target nation on one hand, and on the other hand the full ethical and cultural implication of the health intervention that needs to be implemented.

The discourse on desexualization of vaccines pertains directly to governing multicultural, diverse societies. Desexualization tends to infringe on the values, and ultimately hurt the interests of those segments of society which adhere to a worldview (let us call it, if only for the sake of simplicity, a “Western-Enlightenment” worldview), which highlights instrumental rationality, individualism, utilitarianism, secularism and progress as its foundational values. On the other hand, desexualization may be instrumental in promoting vaccination among societal groups that espouse a more conservative approach, whose understanding of modernity does not necessarily also carries over the Western-Enlightenment values mentioned above.

To provide a concrete example of the dilemmas related to addressing sexual contexts of certain vaccines in a multicultural society we shall now focus on the implementation of HPV vaccination in Israel. We shall address specifically the “secular/religious” tension between the Orthodox Jewish community and the “modern” secular urban Jewish society. Israeli policy-makers can address this conflict by adopting three different approaches: Either adhering to a single, unified approach (i.e., either desexualization or sexualization), or juggling between the two approaches.

One approach would be to develop different vaccination strategies for Orthodox versus secular groups. One could adopt a desexualizing HPV program for the Orthodox Jewish population, vaccinating children at the age of 9 years in this segment of society, thus avoiding an explication of a discourse on sexuality that may not sit well with members of this community. In the same vein, policy makers could adopt a sexualizing HPV program for adolescents in other groups of Israelis, who are more likely to engage in pre-marital sexual activity in their adolescence, and in which community, as a general rule, an open discourse on sexuality is not taboo.

The technical implementation of this policy is rather feasible, yet the public implication could be problematic. Such policy may stir a notable public debate which will draw increased attention to the sexual context of HPV, practically hampering all attempts for desexualization. Moreover, this debate may affect the already loaded interrelationship between Orthodox and non-Orthodox Jews in Israel and contribute to intercultural schisms.

The second approach would be to adhere to a uniform desexualized HPV vaccination strategy (e.g. universal vaccination at the age of 9 years). This could suit a society where the major fraction of the population is conservative, religious, or otherwise tends to view reality through a lens different than that of liberal-Westernized culture. But it would be less suitable in a truly multicultural or diverse society such as Israel. A situation where a developed country will abandon Western principles, such as respect for individual autonomy and transparency, is highly unlikely. In Europe, for example, this would probably ignite the already flammable tension between the native Europeans and immigrants from Asia and Africa.

The third approach would be to adopt a uniform sexualized vaccination strategy, implicating that the state of Israel embraces principles that fit with moral values of governance and the liberal notion of modernity. Groups that traditionally do not adhere to principles entailed in this worldview would have to either adapt, or refrain from vaccination against sexually transmitted disease. Note that this does not mean that the state blindly ignores the uniqueness of minority groups within its population; the decision to adhere to a uniform policy of explicating the sexual aspects of the vaccinations at hand may be the result of a careful, attentive consideration of exactly this uniqueness. The State, to give but one obvious example, could accept– even if only *prima facie –* the claim made by Jewish Orthodox groups that their lifestyle renders HPV vaccination redundant altogether, and not include them from this program (As we noted above, HPV burden among Orthodox Jews in Israel shows this may be a valid claim). This would allow Israeli policymakers to maintain a fully sexualized HPV promotion campaign directed at the secular/liberal or “Westernized” population without being required to engage in a paternalistic interaction with the conservative or religious sector.

Admittedly, our discussion in this example largely follows the prevalent discourse, which designates certain identity groups as outlying the parameters of the wider society, namely the segments commonly identified as “secular” and “religious”. Nevertheless, as we have noted above, it is our contention that sociopolitical reality is vastly more complex than this dichotomous scheme would suggest. A more detailed discussion of the various identity groups and their unique view of the world, of which sexuality is only one part of a larger whole, must be left for future discussions. These would have to deal not only with attitudes towards vaccine sexualization, but also with wider issues of truth, relativism and attitudes towards dominant moral horizons.

### Future challenges

While the debate on sexualization-desexualization of HBV and HPV have not yet been resolved, one can see forthcoming challenges when new vaccines against STI are introduced. Unfortunately, the enormous attempts to develop a vaccine against HIV have failed so far. Nonetheless, we need to be prepared for an eventual success. It is hard to believe that the sexual context of HIV infection would be ignored, and at the same time, it is very likely that policy makers will attempt to introduce the vaccine as part of routine programs and vaccinate the entire population. Given the stigmas associated with HIV, achieving universal vaccination against such a highly sexualized infection will be an immense task.

A new emerging challenge relates to the Zika Virus threat, associated with congenital neurological abnormalities in infants born to mothers infected during pregnancy [68]. The virus is transmitted mainly by Aedes mosquito bites in endemic regions, but more and more evidence is accumulating on sexual transmission through contaminated body fluid [69]. Once a vaccine against Zika is developed, policy makers will need to balance the implications of the sexualization-desexualization of the infection. Here again the debate could become more complicated by stigmas related to the association of sexual transmission with specific nationals (e.g. Latin Americans) or ethnic groups.

Another foreseeable challenge is related to HPV vaccination in the developing world. According to the WHO, over 80% of worldwide deaths from cervical cancer occur in the developing world [70]. The health systems of many countries cannot provide effective treatment, or comprehensive HPV vaccination programs. Even if these shortcomings are solved in the future, the cultural barriers associated with implementing programs to prevent STDs are still expected to affect vaccine acceptance. The challenges related to HPV vaccination in the developing world will have to include addressing cultural variability, as well as relativistic ethical approaches related to sex, autonomy and public engagement.

## Conclusions

At first sight, shaping the sexual framework of vaccination programs may appear to be a practical problem related to promotion strategies. One might claim that vaccine promoters have the prerogative to decide on the profile of the information provided to the public in term of content and formulation, deleting information that is too sensitive, or framing it in a context that is more agreeable to the public. In other words, public health officials have the right to desexualize vaccination programs against STDs. Nevertheless, the thorough analysis of desexualization of vaccines against STIs, provided here, indicates that addressing the sexual context of vaccination is more complicated and value-laden than initially seen, and should be addressed very carefully.

Desexualization of vaccination against HPV invokes complex ethical considerations and underlines the tension between potential utilitarian benefits gained by exercising desexualization and the dangers of infringing on basic ethical principles. It appears that unless very strong evidence is provided that desexualization leads to a substantial decrease in HPV related morbidities, desexualization is actually not justified. In parallel, examination of the sexual implications of HBV vaccination invoke considerations related to the duty of the state to communicate complex, uncomfortable, bio-medical information to the lay public, avoiding unnecessary paternalism.

At the same time, desexualization of vaccines invokes complex societal considerations and underlines the tension between various subgroups in multicultural societies. It must be noted that decisions on the sexual context of vaccines, since related to wider, extra-epidemiological socio-politico-cultural issues, pertain to fundamental challenges and approaches regarding the governing of multicultural and heterogeneous societies. This, as an ever intensifying public debate has proven, is far from being an easily solvable matter, even if narrowed down to fit in with (only) modern Western political philosophy. We must note, however, that the reverse is also true: that a policy of desexualization – that is, of an active repression if not outright disinformation regarding certain diseases and their vaccinations – also sheds light on the way a state views its stance vis-à-vis these political-philosophical considerations.

While this article focuses specifically on the decontextualizing the sexual contexts of certain vaccines, it underlines the importance of truth in conveying health information, which policy makers with primarily disease reduction in mind might be tempted to compromise on. This becomes even more urgent in a political and ethical climate that still questions scientific truths, and more specifically the evidence surrounding vaccinations. All this reflects not only on dealing with the sexual context of vaccination, but also on the way that different stakeholders address the truth related to vaccine safety, which remains the major concern of the public when relating to vaccination.

## Abbreviations

HBV: Hepatitis B Virus
HPV: Human Papilloma Virus
